# Association of Preoperative Circulating Hsp70 Levels with Event-Free Survival in Thoracic Oncology

**DOI:** 10.3390/cancers18193109

**Published:** 2026-09-24

**Authors:** Dominik Lobinger, Nicholas Taylor, Verena Messner, Sophie Seier, Johannes Bodner, Erika Roberts, Ali Bashiri Dezfouli, Alan Graham Pockley, Seyer Safi, Gabriele Multhoff

**Affiliations:** 1 Department of Thoracic Surgery, München Klinik Bogenhausen, Lehrkrankenhaus der Technischen Universität München (TUM), 81925 Munich, Germany; 2 Central Institute for Translational Cancer Research Technische Universität München (TranslaTUM), TUM School of Medicine and Health, TUM Universitätsklinikum, Technische Universität München (TUM), 81675 Munich, Germany; nicholas.taylor@tum.de (N.T.); gabriele.multhoff@tum.de (G.M.); 3 Department of Otolaryngology, Head and Neck Surgery, TUM School of Medicine and Health, TUM Universitätsklinikum, Technische Universität München (TUM), 81675 Munich, Germany; 4 Multimmune GmbH, 81675 Munich, Germany; 5 Division of Thoracic Surgery, TUM School of Medicine and Health, TUM Universitätsklinikum, Technische Universität München (TUM), 81675 Munich, Germany

**Keywords:** thoracic malignancies, NSCLC, Hsp70, biomarker, follow-up

## Abstract

**Objectives/Background**: Heat shock protein 70 (Hsp70) is frequently overexpressed and released from malignant tumors and is associated with tumor aggressiveness and resistance to treatment. While elevated levels of extracellular Hsp70 (eHsp70) in the blood have been associated with unfavorable tumor characteristics, its prognostic significance in the context of long-term follow-up has not yet been sufficiently analyzed. The primary objective of this study was to investigate the prognostic association between preoperative circulating eHsp70 levels and subsequent event-free survival (EFS) in patients with NSCLC undergoing thoracic surgery. Patients with pulmonary metastases from other primary tumors were analyzed as an exploratory subgroup. **Methods**: The association between eHsp70 levels, clinical outcome, and event-free survival in patients with malignant thoracic diseases was investigated in a retrospective study. **Results**: A total of 76 patients undergoing thoracic surgery for non-small cell lung cancer (NSCLC) or pulmonary metastases from other primary tumors were included and subsequently followed for disease progression, metastasis, or death. Preoperative eHsp70 levels were significantly higher in patients who experienced an adverse clinical event than in event-free patients (median 194.3 ng/mL [IQR 19.9–493.0] vs. 27.8 ng/mL [IQR 14.3–53.4], *p* = 0.0064). ROC analysis showed moderate discriminatory performance in the overall cohort (AUC 0.68, 95% CI 0.55–0.81), with an AUC of 0.72 (95% CI 0.56–0.88) in patients with NSCLC and 0.59 (95% CI 0.34–0.83) in patients with pulmonary metastases. In patients with NSCLC, elevated preoperative eHsp70 levels were associated with significantly shorter event-free survival (log-rank *p* < 0.0001). In multivariable Cox regression analysis restricted to patients with NSCLC, eHsp70 remained independently associated with shorter event-free survival after adjustment for tumor stage, age, and sex (HR 1.33, 95% CI 1.07–1.65, *p* = 0.010). **Conclusions**: These findings suggest that circulating eHsp70 may have prognostic relevance in patients with NSCLC. However, prospective validation in larger and independent cohorts is required before its clinical utility can be established.

## Simple Summary

Reliable blood-based biomarkers that may help identify cancer patients at increased risk of disease progression remain an important unmet clinical need. Heat shock protein 70 (Hsp70) is frequently overexpressed and released by tumor cells and has been associated with tumor aggressiveness. In this retrospective study, we investigated whether preoperative circulating extracellular Hsp70 (eHsp70) levels were associated with subsequent clinical outcomes in patients undergoing thoracic surgery for non-small cell lung cancer (NSCLC) or pulmonary metastases. Patients with NSCLC who experienced disease progression, metastasis, or death had higher preoperative eHsp70 levels and shorter event-free survival than patients without an event. In contrast, no statistically significant association with event-free survival was observed in the smaller pulmonary metastasis cohort. In patients with NSCLC, the association between eHsp70 and event-free survival persisted after adjustment for tumor stage, age, and sex. These findings suggest that circulating eHsp70 may have prognostic relevance in NSCLC. However, the retrospective design, small sample size, and heterogeneity of the study population limit the conclusions that can be drawn. Larger prospective studies are needed to validate these findings.

## 1. Introduction

The stress-inducible heat shock protein 70 (Hsp70), which is evolutionarily highly conserved serves as a molecular chaperone to maintain protein homeostasis and support protein folding. Furthermore, it interacts with signaling pathways that influence cell proliferation and differentiation [1]. The expression of Hsp70 is strongly upregulated in response to a large variety of external stressors, including thermal stress and ischemia [2,3]. Hsp70 is overexpressed in a variety of tumor types and is localized not only in the cytosol but also on the plasma membrane of tumor cells, but not on membranes of normal cells [4]. In addition, Hsp70 can be detected extracellularly (eHsp70) in the blood either as a free molecule passively released from dying cells or associated with lipid microvesicles that are actively secreted by viable tumor cells [5]. Hsp70 overexpression has been associated with treatment resistance and tumor aggressiveness in various cancers. Furthermore, the role of Hsp70 in both the development and treatment of tumors has already been discussed extensively [6,7,8,9]. In addition to its diagnostic potential, membrane Hsp70 expressed on tumor cells can exert immunomodulatory functions and has been confirmed as a clinically relevant selective target for ex vivo activated immunocompetent natural killer (NK) cells in Phase I and Phase II clinical trials [10].

Although some studies, as described above, have reported an association between overexpression of Hsp70 and poorer treatment response and survival, data on long-term outcomes remain limited [11,12]. Our recent study demonstrated that high eHsp70 levels in the blood of patients with thoracic tumors prior to any surgery, primarily non-small cell lung cancer (NSCLC), are associated with more advanced tumor stages, the presence of lymph node metastases, and an increased risk of early recurrence [13]. In this retrospective analysis, a long-term follow-up of patient outcomes was conducted.

Reliable blood-based biomarkers for risk stratification and disease monitoring remain an unmet clinical need in thoracic oncology [14,15]. Although established clinicopathological factors, such as tumor stage, provide important prognostic information, additional biomarkers could improve the identification of patients with an increased risk of recurrence or disease progression during follow-up [16]. Especially in an era of increasingly complex treatment options and the emergence of neoadjuvant and perioperative regimens, it is essential to establish prognostic biomarkers to identify which patient groups are most likely to benefit from which treatment. The landscape of therapeutic approaches has expanded rapidly in recent years. Thanks to innovative immunotherapies and targeted therapies, the prognosis for patients with NSCLC has improved, in some cases significantly [17,18,19,20,21]. Given the biological role of Hsp70 in tumor progression and treatment resistance, levels of circulating eHsp70 represent a potential biomarker candidate for predicting disease course [22,23].

This study further investigated the prognostic significance of eHsp70 levels prior to therapy in patients with different thoracic malignancies during long-term follow-up. Specifically, we examined whether preoperatively defined eHsp70 levels were associated with clinical outcomes and event-free survival in a retrospective cohort of patients undergoing routine oncological follow-up. Therefore, the primary objective of the present study was to investigate whether preoperative circulating eHsp70 levels are associated with subsequent event-free survival in patients with NSCLC undergoing thoracic surgery. We hypothesized that higher preoperative eHsp70 levels would be associated with shorter event-free survival. Patients with pulmonary metastases from other primary tumors were included as an exploratory subgroup to assess whether the observed association was also present across other thoracic malignancies.

## 2. Materials and Methods

### 2.1. Study Population

The retrospective follow-up analysis included patients whose eHsp70 levels had been measured at a single preoperative time point as part of one of our previous studies prior to a planned thoracic surgical procedure. Surgery was either performed for the treatment of NSCLC or for pulmonary metastasectomy in patients with other primary malignancies. The primary endpoint was event-free survival (EFS), defined as the time from surgery to disease progression, development of new metastases, or death.

Clinical endpoints were defined as disease progression, the development of new metastases, or death. The study was approved by the local ethics committee of the TUM School of Medicine and Health (ethical approval number: 2403/09 and 2428/09), and written informed consent was obtained from all patients and healthy donors before the start of the study. The study was conducted in accordance with the guidelines of the Declaration of Helsinki.

### 2.2. Hsp70-Exo Sandwich ELISA

Circulating extracellular Hsp70 (eHsp70) levels were measured using the Hsp70-exo sandwich ELISA according to the protocol established and validated in our previous studies. The assay has been successfully applied in patients with thoracic malignancies and other solid tumors. Detailed descriptions of blood sampling procedures, sample processing, assay characteristics, and analytical validation have been published previously [13]. Blood samples were collected under non-fasting conditions. Plasma was obtained from EDTA-anticoagulated blood by centrifugation at 1500× *g* for 15 min at room temperature, aliquoted (300 µL), and stored at −80 °C. Samples underwent no more than one freeze–thaw cycle before Hsp70 analysis.

### 2.3. Statistical Analysis

Continuous variables are presented as median and interquartile range (IQR). Differences in eHsp70 levels between patients with and without adverse clinical events were assessed using the Wilcoxon rank-sum test. Receiver operating characteristic (ROC) analysis was performed to evaluate the discriminatory ability of Hsp70 and to determine the optimal cutoff value using the Youden index. The cutoff was determined from the Youden index in the respective analysis cohort and was therefore data-driven rather than prespecified or based on an established clinical threshold. Kaplan–Meier analyses were subsequently performed using this ROC-derived cutoff. Event-free survival (EFS) was analyzed using the Kaplan–Meier method and compared by the log-rank test. Cox proportional hazards regression models were used to estimate hazard ratios (HRs) and 95% confidence intervals (CIs). Hsp70 was analyzed as a continuous variable after log2 transformation, allowing interpretation of HRs per doubling of Hsp70 levels. Multivariable Cox regression was performed in the NSCLC subgroup and adjusted for disease stage (I–II vs. III–IV), age and sex. Statistical analyses were performed using R-Studio version 4.5.2. *p*-values < 0.05 were considered statistically significant.

## 3. Results

### 3.1. Patient Characteristics

Medium- to long-term postoperative follow-up data from routine oncological surveillance were analyzed from 76 patients whose eHsp70 levels had been determined one to two days before thoracic surgery. The cohort comprised 53 patients with non-small cell lung cancer (NSCLC) and 23 patients with pulmonary metastases originating from other primary tumors. Among the 53 patients with NSCLC, 19 had stage I disease, 11 had stage II disease, 13 had stage III disease, and 10 had stage IV disease. The cohort consisted of 34 women and 42 men with an average age of 73 years. The pulmonary metastasis cohort comprised 23 patients with heterogeneous primary tumor entities. The most frequent primary tumor was colorectal cancer (n = 5), followed by squamous cell carcinoma (n = 2), prostate cancer (n = 2), and urothelial carcinoma (n = 2). The remaining patients had various primary malignancies, each represented by one patient. The NSCLC cohort comprised 53 patients, including 30 with adenocarcinoma, 12 with squamous cell carcinoma, while the remaining 11 patients had other histological entities (Supplementary Table S1). Detailed patient characteristics are summarized in Table 1 (detailed information about comorbidities in Supplementary Table S2). eHsp70 levels were measured between 2021 and 2023, and the postoperative follow-up data were then analyzed up to the time of manuscript submission. More details on the postoperative treatment regimens are presented in Supplementary Table S3.

### 3.2. Association of Hsp70 with Clinical Outcome

Preoperative eHsp70 levels were significantly higher in patients who subsequently experienced progression, metastasis, or death than in event-free patients in the NSCLC cohort (236.9 [IQR 28.7–464.4] vs. 27.0 [IQR 13.1–55.4] ng/mL; *p* = 0.0084; Figure 1A). In contrast, no significant difference in eHsp70 levels was observed between patients with and without adverse clinical outcomes in the pulmonary metastasis cohort (68.3 [IQR 19.9–580.5] vs. 28.6 [IQR 22.9–39.3] ng/mL; *p* = 0.5083; Figure 1B). Within the NSCLC cohort, median eHsp70 levels were 35.4 ng/mL in patients with stage I–II disease and 54.4 ng/mL in those with stage III–IV disease, with no statistically significant difference between the groups (*p* = 0.2695).

Receiver operating characteristic (ROC) analysis of the overall cohort showed moderate discriminatory performance of preoperative circulating eHsp70 levels for adverse clinical outcomes, with an area under the curve (AUC) of 0.68 (95% CI 0.55–0.81; Figure 2A). In the NSCLC cohort, the AUC was 0.72 (95% CI 0.56–0.88; Figure 2B). The optimal Youden-derived cutoff was 168.86 ng/mL, corresponding to a sensitivity of 60.0% and specificity of 87.9%. In the pulmonary metastasis cohort, discriminatory performance was lower (AUC 0.59, 95% CI 0.34–0.83), with a sensitivity of 50.0% and specificity of 88.9% at a cutoff of 75.95 ng/mL. Given the small sample size and wide confidence interval, these cohort-specific estimates should be considered exploratory. Kaplan–Meier analysis demonstrated significantly shorter event-free survival among patients with NSCLC and elevated preoperative eHsp70 levels, defined by the cohort-specific optimal cutoff of 168.86 ng/mL, compared with those with lower eHsp70 levels (log-rank *p* < 0.0001; Figure 3A). In contrast, among patients with pulmonary metastases, event-free survival did not differ significantly according to preoperative eHsp70 levels using the cohort-specific cutoff of 75.95 ng/mL (log-rank *p* = 0.0804; Figure 3B). In two patients, circulating Hsp70 was additionally measured during postoperative follow-up. In one patient, a Hsp70 concentration of 2.32 ng/mL was measured 3 months after surgery, and the patient remained clinically unremarkable during follow-up. In a second patient, Hsp70 was measured 6 months after surgery and was markedly elevated at 597.74 ng/mL, coinciding with documented disease progression. Given the very limited number of postoperative measurements and the different sampling times, these observations were considered descriptive only.

In univariable Cox analysis of the overall cohort, each doubling of the eHsp70 con-centration was associated with a 25% increase in the risk of adverse events (HR 1.25 per doubling of eHsp70, 95% CI 1.09–1.44, *p* = 0.002). In the NSCLC subgroup (n = 53, 20 events), analyzed by multivariable Cox regression analysis including tumor stage, age, and sex, elevated circulating eHsp70 levels remained independently associated with an increased risk of an adverse event (Figure 4). Each doubling of the preoperative eHsp70 level was associated with a 33% increase in risk (HR 1.33, 95% CI 1.07–1.65, *p* = 0.010). Tumor stage (HR 1.98, 95% CI 0.72–5.42, *p* = 0.186), age (HR 1.03, 95% CI 0.97–1.10, *p* = 0.349), and sex (HR 0.99, 95% CI 0.39–2.51, *p* = 0.984) were not independently associated with event-free survival. Detailed results of the univariable and multivariable Cox regression analyses are provided in Supplementary Table S4.

## 4. Discussion

Our retrospective cohort study demonstrates that elevated eHsp70 levels that are present prior to treatment are associated with adverse clinical outcomes and reduced event-free survival in patients with malignant thoracic diseases, particularly those with NSCLC. Patients who experienced disease progression, metastasis, or death during the follow-up period had significantly higher baseline eHsp70 levels compared to event-free patients. Furthermore, elevated eHsp70 levels were associated with shorter event-free survival in the Kaplan–Meier analysis. It is noteworthy that this association persisted in the NSCLC subgroup even after adjusting for tumor stage, age and sex in the multivariate Cox regression analysis, with eHsp70 remaining independently associated with poor outcomes. These results are consistent with our previous observations, which showed that elevated eHsp70 levels in patients with NSCLC were linked to advanced tumor stage, lymph node involvement, and early recurrence [13].

While previous analyses focused primarily on tumor burden and short-term clinical outcomes, the present study shows that baseline eHsp70 levels before surgery already provide prognostic information over longer follow-up periods. Our findings are consistent with previous reports linking elevated eHsp70 expression to more aggressive tumor biology, treatment resistance, and poor clinical outcomes across various tumor types [24,25]. The potential prognostic significance of extracellular Hsp70 is further supported by observations in other tumor types. In a cohort of patients with non-metastatic breast cancer, gradually rising eHsp70 was observed during therapy in two patients who subsequently experienced disease recurrence, whereas eHsp70 remained stable in patients who did not later experience treatment failure. Together with our findings, this supports further investigation of circulating eHsp70 as a potential biomarker of clinical outcome in oncology [26].

Several biological mechanisms may explain the observed association between elevated eHsp70 levels and adverse disease outcomes.

Apart from its well-known role as a molecular chaperone, Hsp70 performs several tumor-promoting functions that could explain its association with poor clinical outcomes. It has been shown that elevated Hsp70 expression promotes tumor cell survival by stabilizing target proteins involved in tumor growth and protecting cancer cells from cellular stress. Furthermore, Hsp70 inhibits several apoptotic pathways, thereby promoting the survival of tumor cells during disease progression and metastasis. Several studies have demonstrated a link between elevated Hsp70 expression and increased metastatic potential, including enhanced invasion, migration, and vascular infiltration. Hsp70 has also been linked to the epithelial–mesenchymal transition (EMT), cell motility, and extracellular matrix remodeling—processes that are critical for metastatic spread. Extracellular Hsp70 may also contribute to the development of a pro-inflammatory and metastasis-promoting tumor microenvironment by promoting angiogenesis and modulating the activity of matrix metalloproteinases [4,27]. Taken together, Hsp70 supports tumor cell survival under conditions of cellular stress and contributes to the stabilization of proteins involved in proliferation and anti-apoptotic signaling pathways. Furthermore, viable tumor cells actively release Hsp70-containing extracellular vesicles, suggesting that circulating eHsp70 levels may reflect tumor activity and biological aggressiveness. Consequently, elevated eHsp70 concentrations could serve as a surrogate marker of increased tumor burden or treatment-resistant disease. It should be noticed that circulating Hsp70 is not exclusively tumor-derived and may also be influenced by systemic conditions. Inflammatory processes, infections, tissue injury, cellular stress, and cardiovascular or other comorbid conditions may contribute to circulating Hsp70 levels.

In addition, eHsp70 can also act as a damage-associated molecular pattern (DAMP), thereby triggering the activation of the immune cascade. This activation is mediated, among other things, by the engagement of TLR2 and TLR4 receptors and the resulting increased release of inflammatory mediators [28]. Although membrane-bound Hsp70 acts as a potential target for NK cells, the antitumor activity of NK cells is frequently suppressed in advanced stage tumor patients. Elevated eHsp70 levels in the circulation may also indicate ongoing tumor–host interactions which are associated with disease progression [29,30]. In a Phase II clinical trial, the administration of autologous NK cells stimulated ex vivo with an Hsp70-derived peptide (TKD) and low-dose IL-2, which stimulates NK cells to target membrane Hsp70, was associated with improved clinical outcomes in patients with advanced non-small cell lung cancer (NSCLC) [10]. These results underscore once again that elevated Hsp70 levels reflect clinically significant interactions between tumor environment and immune system and should be further investigated in the context of personalized immunotherapy strategies.

Recent findings suggest interactions between Hsp70-associated immune pathways and signaling at immune checkpoints, including PD-1/PD-L1-mediated immune evasion mechanisms, which could influence the response to immunotherapy. Consequently, elevated levels of eHsp70 in the blood may not only reflect tumor burden and cellular stress but could also reflect changes within the tumor’s immunological microenvironment. Since no immunological parameters were available in our retrospective cohort, future prospective studies are needed that combine measurements of circulating eHsp70 with immunoprofiling to further elucidate these mechanisms in malignant thoracic diseases [31,32,33,34].

The ROC analysis showed that eHsp70, with an AUC value of 0.68, has moderate discriminatory power in predicting adverse clinical outcomes. Although this performance is insufficient for use as a standalone prognostic marker, eHsp70 nevertheless may provide clinically relevant prognostic information when combined with established clinicopathological factors such as tumor stage. The persistence of the association after adjustment for tumor stage, age, and sex suggests that circulating eHsp70 may provide additional prognostic information beyond conventional staging in patients with NSCLC. The ROC analyses should be interpreted cautiously. In the overall cohort, preoperative circulating eHsp70 showed only moderate discrimination for event status (AUC 0.68, 95% CI 0.55–0.81). Although discrimination was higher in the NSCLC subgroup (AUC 0.72, 95% CI 0.56–0.88), these findings do not support the use of eHsp70 as a standalone predictive biomarker. Rather, the ROC results indicate a potential association with outcome that requires confirmation in larger and independent cohorts and should be evaluated in combination with established clinical and pathological prognostic factors. In patients with pulmonary metastases, the discriminatory performance of circulating eHsp70 was limited (AUC 0.59, 95% CI 0.34–0.83). Several factors may contribute to this finding. First, the pulmonary metastasis subgroup was small (n = 23), with only 14 events, resulting in considerable statistical uncertainty, as reflected by the wide confidence interval. Second, this subgroup comprised metastases originating from different primary tumor types and therefore represents a biologically heterogeneous population. Differences in tumor biology, tumor burden, and the propensity for Hsp70 release among different tumor entities may influence circulating eHsp70 concentrations and their relationship with clinical outcome. Thus, the weaker discriminatory performance in this subgroup may reflect both limited statistical power and biological heterogeneity. Given the small sample size and heterogeneous tumor origins, however, these explanations cannot be distinguished reliably within the present cohort.

Our findings are consistent with previous studies demonstrating a role for Hsp70 in malignant thoracic diseases. Elevated Hsp70 expression has been associated with tumor progression in patients with non-small cell lung cancer (NSCLC) [13]. Our long-term follow-up data complement these observations by showing that elevated preoperative eHsp70 levels in the blood are associated with significantly shorter event-free survival and remain independently associated with poor clinical outcomes in patients with non-small-cell lung cancer (NSCLC).

Pulmonary metastases represent a biologically heterogeneous entity that differs significantly from non-small cell lung cancer (NSCLC) in terms of tumor biology, metastatic behavior, and treatment strategies. Consequently, circulating eHsp70 may reflect both common stress-associated mechanisms shared by all tumor entities and tumor-specific biological processes. While our previous findings support the concept of circulating eHsp70 as a cancer-transcending stress biomarker, the present data particularly highlight its prognostic relevance in patients with NSCLC.

Furthermore, serial measurements of eHsp70 and the assessment of its dynamics may provide additional insights into the development of the tumor burden and treatment response. If confirmed in larger prospective cohorts, eHsp70 could complement established staging systems for risk stratification during follow-up.

There are several limitations to this study that should be noted. First, it was a retrospective study conducted at a single center, which could limit the generalizability of the results. Second, the sample size was relatively small, particularly for the subgroup analyses with heterogenous patients, resulting in limited statistical power. In addition, external validation in larger, independent cohorts is essential before clinically applicable sensitivity, specificity, and predictive values can be determined. Third, the cohort included different types of malignant thoracic tumors, resulting in biological heterogeneity. Furthermore, the eHsp70 cutoff used for Kaplan–Meier stratification was derived from ROC analysis within the present cohort and subsequently evaluated in the same population. This data-driven approach may lead to optimistic estimates of discriminatory performance and survival separation. The identified threshold should therefore be considered exploratory and requires validation in independent cohorts, ideally using prespecified thresholds. In addition, the generalizability of the present findings is also limited by the surgical nature of the study population. Patients included in this cohort underwent thoracic surgery and therefore represent a selected population with disease considered amenable to surgical treatment. Whether the prognostic association observed here is also applicable to nonsurgical patient populations remains to be determined in prospective studies including broader and clinically diverse NSCLC cohorts. Finally, only one pre-operative eHsp70 level was available, precluding the assessment of whether longitudinal changes in eHsp70 levels provide additional prognostic information during follow-up. Therefore, consecutive eHsp70 measurements at various time points during and after therapy—taking into account different adjuvant therapy regimens—would be a good starting point for further studies. Since treatment-related factors significantly influence event-free survival and may interact with circulating eHsp70 levels, future prospective studies incorporating detailed treatment information are needed to more precisely define the prognostic value of circulating eHsp70 in malignant thoracic diseases. Taken together, this analysis serves as a pilot study to investigate the association between eHsp70 levels and long-term survival and oncological outcomes.

As mentioned above, this study focuses primarily on the prognostic significance of a single preoperative measurement of circulating eHsp70 levels. Since eHsp70 was generally measured only before surgery, no conclusions can be drawn from the study regarding postoperative changes or the benefits of longitudinal monitoring. The two available postoperative measurements provide only descriptive observations: In a clinically unremarkable patient, a low Hsp70 level was detected 3 months after surgery, whereas in a patient with concurrently documented disease progression, a significantly elevated level was measured 6 months after surgery. Due to the very small number of measurements and the varying postoperative time points, no prognostic or predictive correlations can be derived from these observations. Nevertheless, they illustrate the potential utility of serial eHsp70 measurements in assessing whether changes over time are associated with disease recurrence, disease progression, or response to treatment. Prospective studies with standardized longitudinal samples taken before surgery and at predefined postoperative and treatment-related time points are therefore warranted.

In summary, elevated eHsp70 levels in patients with malignant thoracic tumors could be associated with adverse clinical outcomes and shorter event-free survival. These findings support the potential role of circulating eHsp70 as a prognostic biomarker for risk stratification during oncological follow-up. Larger prospective studies are needed to validate these observations and further define the clinical utility of eHsp70 in thoracic oncology.

## 5. Conclusions

In summary, higher preoperative levels of eHsp70 in the blood were associated with shorter event-free survival in patients with non-small cell lung cancer (NSCLC) who underwent thoracic surgery. These findings support its potential as a prognostic biomarker candidate. However, eHsp70 should not yet be considered an established standalone clinical biomarker. Independent, prospective studies with sufficient statistical power, using standardized Hsp70 assays, predefined cut-off values, and longitudinal measurements, are needed to validate these findings and determine whether eHsp70 provides clinically relevant prognostic information beyond established clinical–pathological factors in patients with thoracic malignancies.

## Figures and Tables

**Figure 1:**
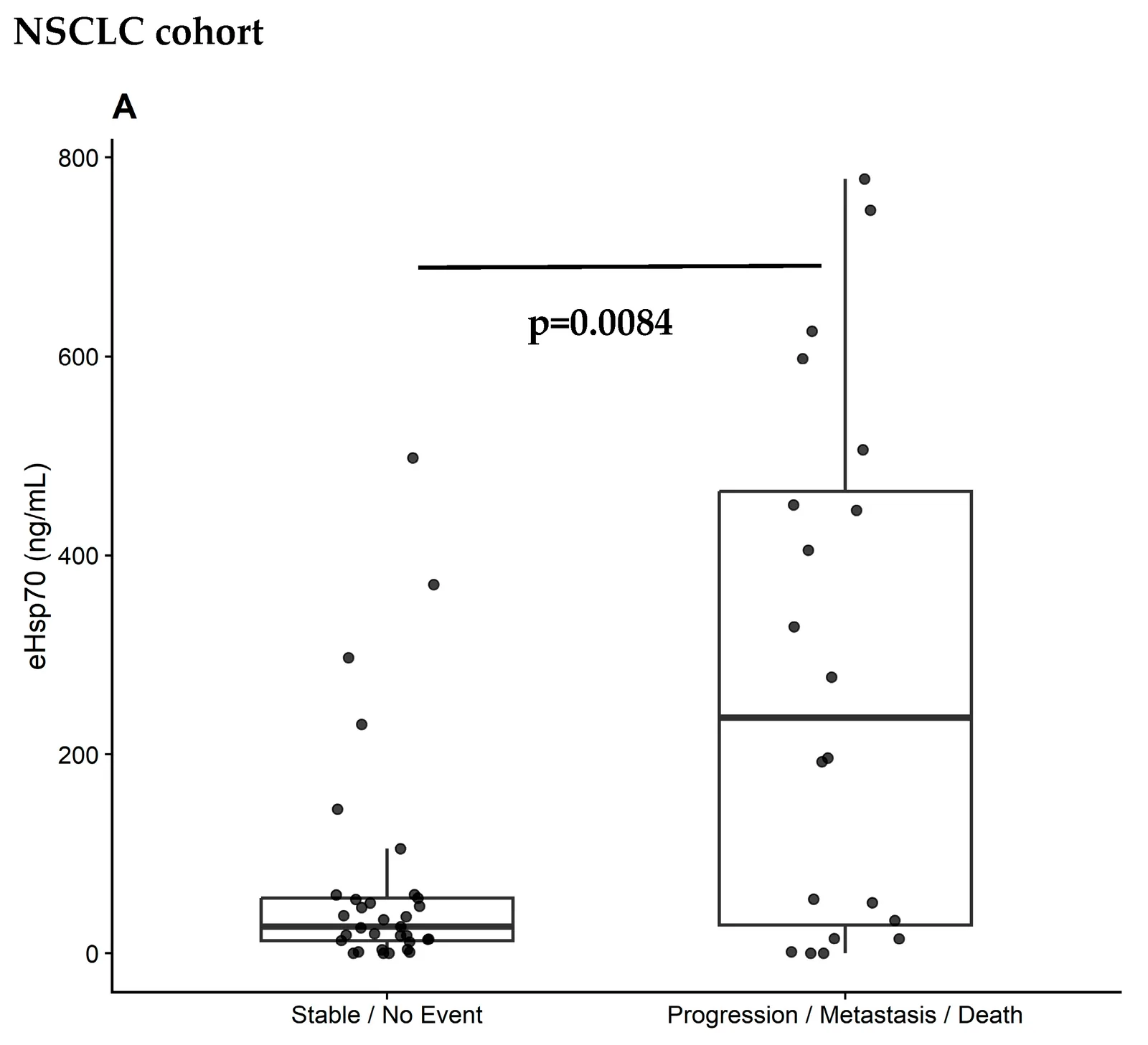

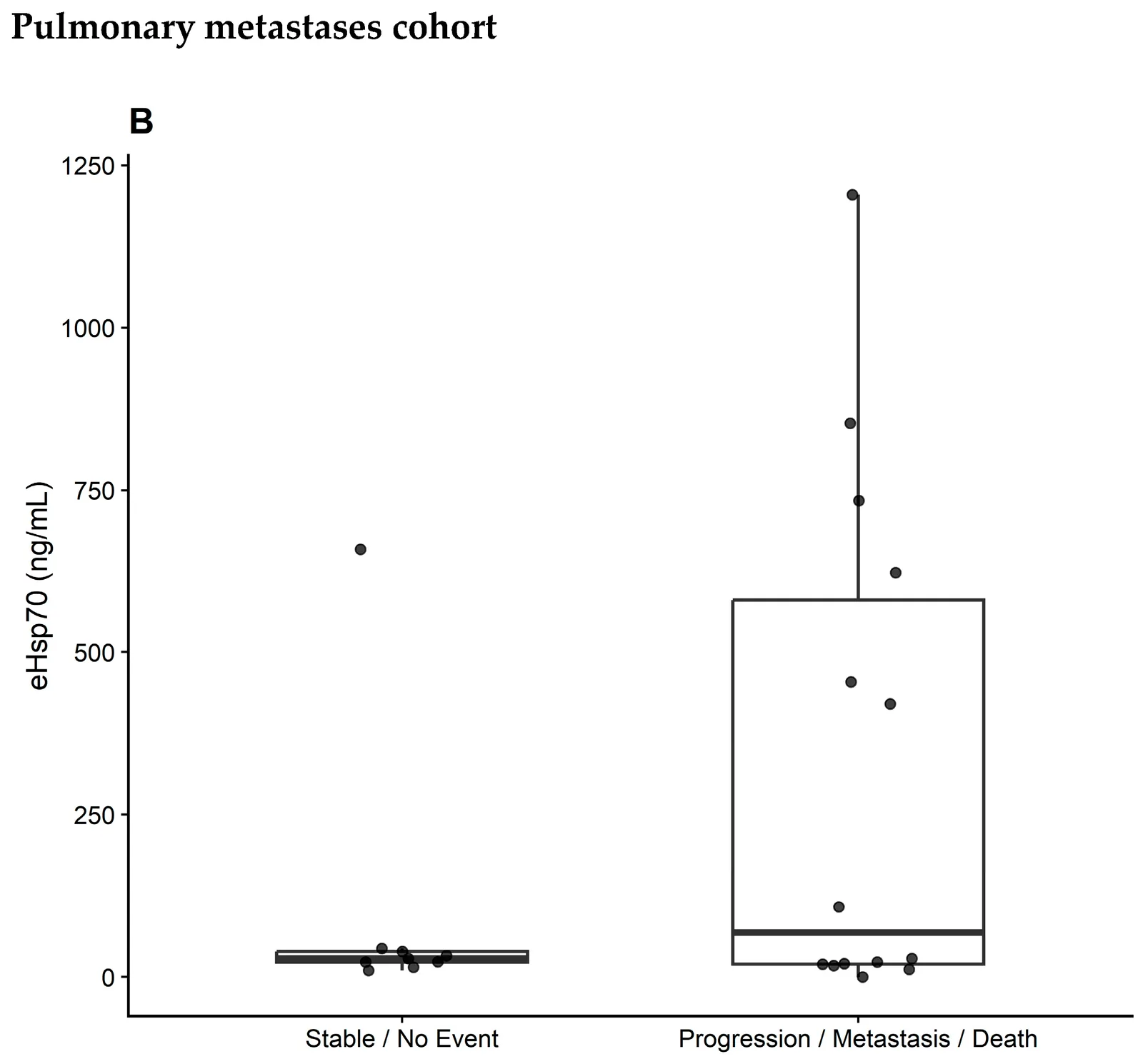
Preoperative circulating eHsp70 levels according to clinical outcome. (**A**) Preoperative circulating eHsp70 levels in patients with NSCLC stratified according to clinical outcome. Patients who experienced progression, metastasis, or death had significantly higher eHsp70 levels than patients without an event (median 236.9 ng/mL [IQR 28.7–464.4] vs. 27.0 ng/mL [IQR 13.1–55.4], *p* = 0.0084). (**B**) Preoperative circulating eHsp70 levels in patients undergoing pulmonary metastasectomy stratified according to clinical outcome. No significant difference was observed between patients with and without an event (68.3 ng/mL [IQR 19.9–580.5] vs. 28.6 ng/mL [IQR 22.9–39.3], *p* = 0.5083). Individual data points are shown together with boxplots indicating the median and interquartile range. Statistical comparisons were performed using the Wilcoxon rank-sum test.

**Figure 2:**
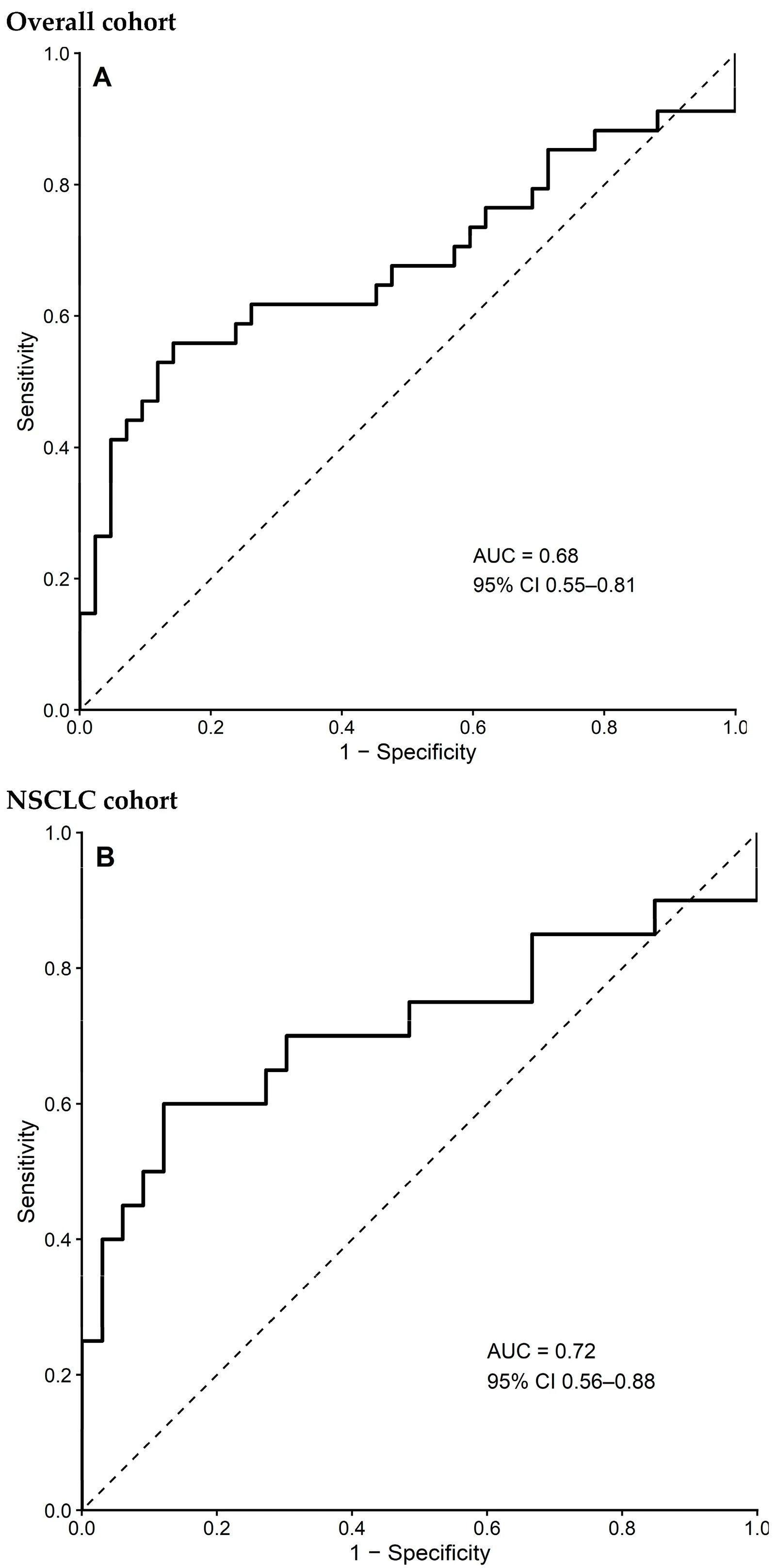
Receiver operating characteristic (ROC) analysis of preoperative circulating eHsp70 levels. (**A**) ROC analysis for the overall cohort of patients with malignant thoracic diseases, showing an area under the curve (AUC) of 0.68 (95% CI 0.55–0.81). (**B**) ROC analysis for the NSCLC cohort, showing an AUC of 0.72 (95% CI 0.56–0.88). The optimal Youden-derived cutoff for the NSCLC cohort was 168.86 ng/mL, with a sensitivity of 60.0% and specificity of 87.9%. ROC analysis of the pulmonary metastasis cohort yielded an AUC of 0.59 (95% CI 0.34–0.83) and is reported in the text because of the small cohort size. The diagonal dashed line represents no discriminatory ability. AUC, area under the curve; CI, confidence interval; eHsp70, extracellular heat shock protein 70; NSCLC, non-small cell lung cancer.

**Figure 3:**
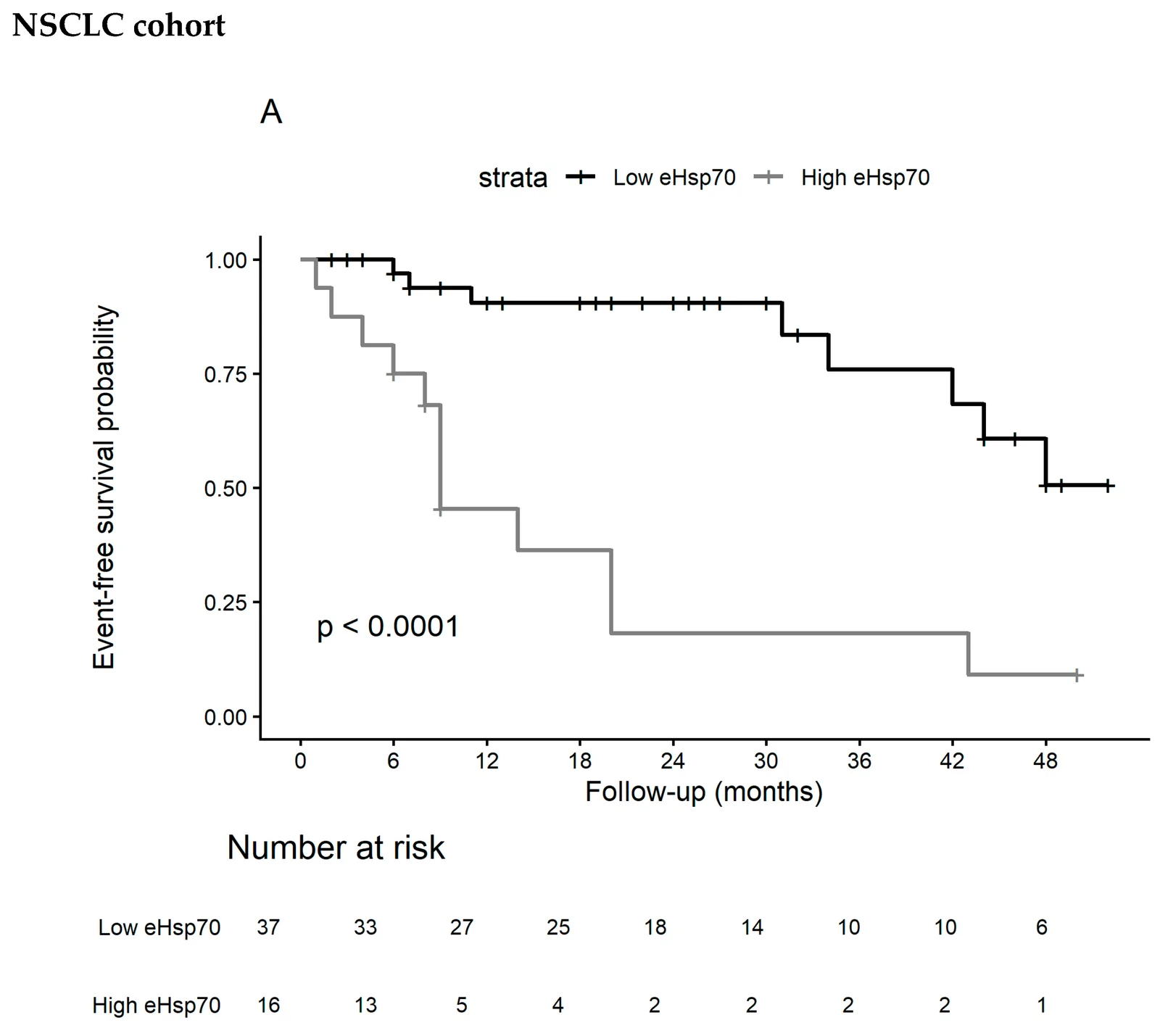

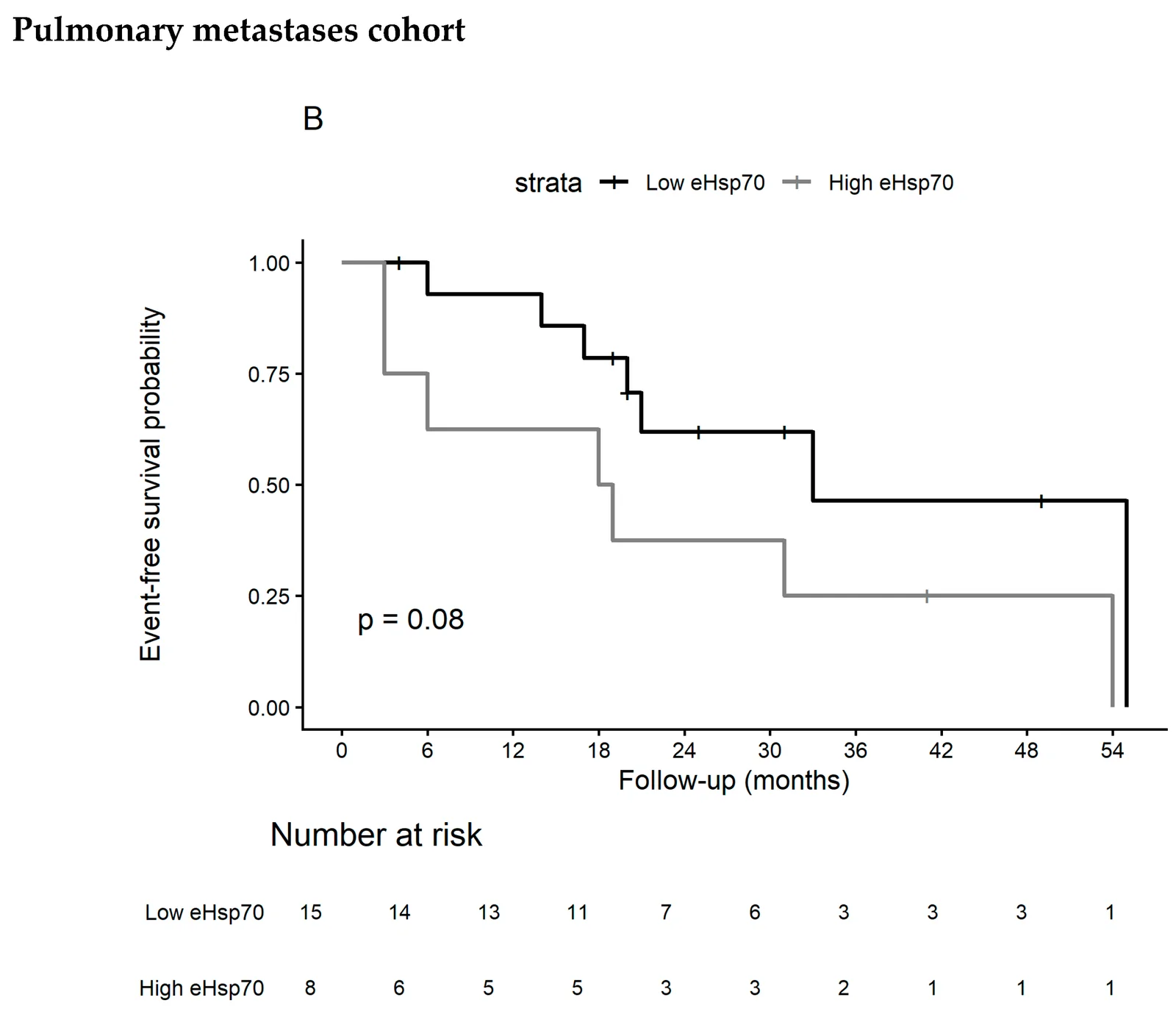
Event-free survival according to preoperative circulating eHsp70 levels. (**A**) Kaplan–Meier analysis of event-free survival in patients with NSCLC stratified according to preoperative circulating eHsp70 levels using the cohort-specific optimal Youden-derived cutoff of 168.86 ng/mL. Patients with elevated eHsp70 levels had significantly shorter event-free survival than those with lower levels (log-rank *p* < 0.0001). (**B**) Kaplan–Meier analysis of event-free survival in patients with pulmonary metastases stratified according to preoperative circulating eHsp70 levels using the cohort-specific optimal Youden-derived cutoff of 75.95 ng/mL. No statistically significant difference in event-free survival was observed between the eHsp70 groups (log-rank *p* = 0.0804). Curves represent event-free survival probability; tick marks indicate censored observations. Numbers at risk are shown below each Kaplan–Meier curve. eHsp70, extracellular heat shock protein 70; NSCLC, non-small cell lung cancer.

**Figure 4:**
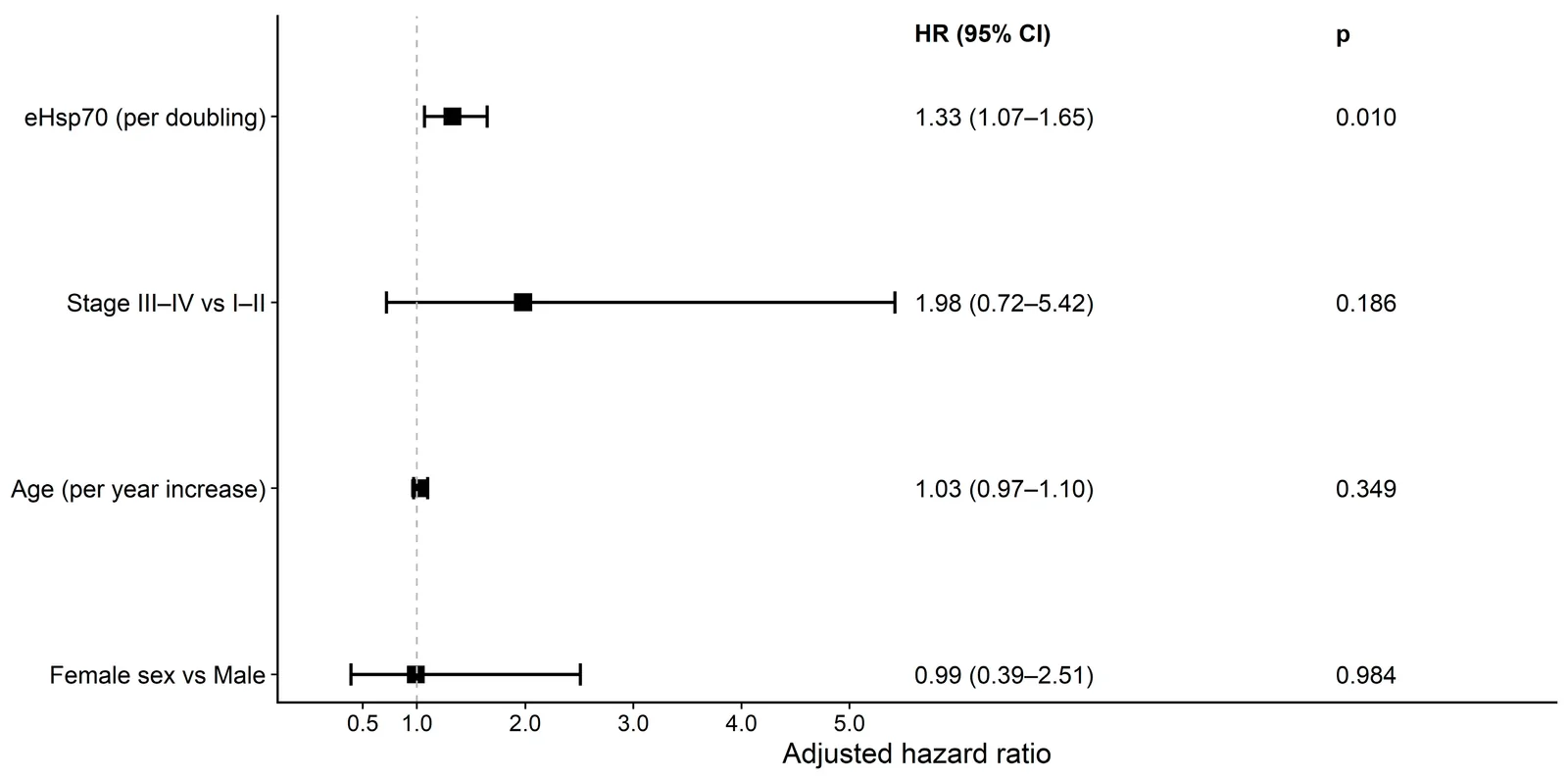
Multivariable Cox regression analysis for event-free survival in patients with NSCLC. Hazard ratios were adjusted for tumor stage, age, and sex. Elevated circulating eHsp70 levels remained independently associated with reduced event-free survival after multivariable adjustment (HR 1.33, 95% CI 1.07–1.65, *p* = 0.010). The analysis included all 53 patients with NSCLC.

**Table 1:** Baseline characteristics of the study cohort stratified by clinical outcome. Patients were classified according to the occurrence of progression, metastasis, or death during follow-up. Continuous variables are presented as median (interquartile range, IQR) and categorical variables as number (%).

Characteristic	No Event (n = 42)	Event (n = 34)
Age, years, median (IQR)	73.5 (64–80)	74.0 (68–79)
Female sex, n (%)	19 (45.2)	15 (44.1)
eHsp70, ng/mL, median (IQR)	27.8 (14.3–53.4)	194.3 (19.9–493.0)
NSCLC, n (%)	33 (78.6)	20 (58.8)
Pulmonary metastases, n (%)	9 (21.4)	14 (41.2)
NSCLC stage I, n	18	1
NSCLC stage II, n	5	6
NSCLC stage III, n	6	7
NSCLC stage IV, n	4	6
NSCLC histology: adenocarcinoma, n	18	12
NSCLC histology: squamous cell carcinoma, n	8	4
NSCLC histology: other, n	7	4
Relevant comorbidity: arterial hypertension, n	6	8
Relevant comorbidity: COPD, n	7	2
Relevant comorbidity: cardiovascular disease, n	8	3
Relevant comorbidity: hyperlipidemia, n	3	2
Relevant comorbidity: hypothyroidism, n	3	3
Relevant comorbidity: diabetes mellitus, n	2	2
Relevant comorbidity: thromboembolic disease, n	2	1
Relevant comorbidity: autoimmune/inflammatory disease, n	4	2
Relevant comorbidity: chronic kidney disease, n	0	1
Relevant comorbidity: obesity, n	1	1
Relevant comorbidity: interstitial lung disease, n	0	1
Relevant comorbidity: anemia, n	2	0
Postoperative adjuvant therapy in NSCLC, n	17	9
No postoperative adjuvant therapy in NSCLC, n	15	9
Postoperative therapy information missing, n	1	2

## Data Availability

The datasets generated and analyzed during the current study are available from the corresponding author upon reasonable request.

## Supplementary Materials

The following supporting information can be downloaded at: https://www.mdpi.com/article/10.3390/cancers18193109/s1, Table S1. Details on the respective histopathologies; Table S2. Detailed list of comorbidities; Table S3. Postoperative treatment regimens; Table S4. Results of the univariable and multivariable Cox regression analyses.
