# Supplementary material for: Association of Preoperative Circulating Hsp70 Levels with Event-Free Survival in Thoracic Oncology
Source: Cancers (Basel). 2026 Sep 24;18(19):3109. doi: 10.3390/cancers18193109 (PMC13664327; doi:10.3390/cancers18193109)
Supplement: Supplementary file 1 [file cancers-18-03109-s001.zip › cancers-4567016-supplementary.pdf]

cancers

MDPI

Table S1. Details on the respective histopathologies.

Cohort	Histology / primary tumor entity	n	%
NSCLC (n = 53)	Adenocarcinoma	30	56.6
	Squamous cell carcinoma	12	22.6
	Other	11	20.8
Pulmonary metastases (n = 23)	Colorectal cancer	5	21.7
	Squamous cell carcinoma	2	8.7
	Prostate cancer	2	8.7
	Urothelial carcinoma	2	8.7
	Other primary malignancies	12	52.2

Table S2. Detailed list of comorbidities.

Category	Exact documented conditions / examples	n patients
Arterial hypertension	Arterielle Hypertonie; benigne essentielle Hypertonie; art. Hypertonus	14
COPD / chronic obstructive pulmonary disease	COPD GOLD A; COPD GOLD II A; COPD GOLD III; COPD with emphysema; COPD	9
Cardiovascular disease	Paroxysmal atrial fibrillation; AV block III; pacemaker/CRT-P; coronary artery disease; prior NSTEMI/myocardial infarction; dilative cardiomyopathy; mitral valve insufficiency; aortic valve stenosis status post TAVI; aortic aneurysm; carotid atheromatosis; TIA/prior stroke; right bundle branch block/left anterior hemiblock	9
Hyperlipidemia	Hyperlipidämie; Hypercholesterinämie	5
Hypothyroidism	Hypothyreose; substitutionspflichtige Hypothyreose	6
Diabetes mellitus	Diabetes mellitus Typ 2; Diabetes mellitus Typ II; NIDDM Typ 2	4
Thromboembolic disease	Prior leg vein thrombosis; venous thrombosis associated with port; pulmonary artery embolism	3
Autoimmune / inflammatory disease	Rheumatoid arthritis; psoriasis; multiple sclerosis; scleroderma; immune-mediated pneumonitis	6
Chronic kidney disease	Chronische Niereninsuffizienz CKD G4	1
Obesity	Adipositas; Adipositas permagna	2

Interstitial lung disease	Interstitielle Lungenerkrankung (UIP pattern)	1
Anemia	Microcytic hypochromic anemia / suspected anemia of chronic disease; hypochromic normocytic anemia with reduced transferrin saturation	2
Chronic hepatobiliary / pancreatic disease	Steatosis hepatis; suspected liver cirrhosis; chronic pancreatitis / pancreatic pseudocysts	3
Neurologic disorders	Trigeminal neuralgia; epilepsy/seizures; restless legs syndrome; chronic dizziness; chronic back pain / prior disc prolapse	5

Table S3. Postoperative treatment regimens.

Treatment category	n (%)
No postoperative adjuvant therapy	24 (45.3)
Chemotherapy	14 (26.4)
Immunotherapy	3 (5.7)
Chemoimmunotherapy	3 (5.7)
Radiotherapy	3 (5.7)
Chemotherapy + radiotherapy	1 (1.9)
Chemoimmunotherapy + radiotherapy	1 (1.9)
Targeted therapy	1 (1.9)
Neoadjuvant chemoimmunotherapy only	1 (1.9)
BSC	1 (1.9)
Missing	1 (1.9)

Table S4. Results of the univariable and multivariable Cox regression analyses.

Variable	HR	95% CI	p
**Univariable: eHsp70 per doubling, all**	**1.25**	**1.09–1.44**	**0.002**
**Multivariable: eHsp70 per doubling, NSCLC**	**1.33**	**1.07–1.65**	**0.010**
Stage III–IV vs. I–II	1.98	0.72–5.42	0.186
Age, per year	1.03	0.97–1.10	0.349
Female vs. Male	0.99	0.39–2.51	0.984

Disclaimer/Publisher's Note: The statements, opinions and data contained in all publications are solely those of the individual author(s) and contributor(s) and not of MDPI and/or the editor(s). MDPI and/or the editor(s) disclaim responsibility for any injury to people or property resulting from any ideas, methods, instructions or products referred to in the content.

https://doi.org/10.3390/cancers18193109
